# Antifungal characterizations of a novel endo-β-1,6-glucanase from *Flavobacterium* sp. NAU1659

**DOI:** 10.1007/s00253-024-13269-1

**Published:** 2024-08-12

**Authors:** TingTing Xie, Jiming Shen, Zhitao Geng, Fan Wu, Yiwei Dong, Zhongli Cui, Yongheng Liang, Xianfeng Ye

**Affiliations:** Key Laboratory of Agricultural Environmental Microbiology of Ministry of Agriculture and Rural Affairs, Nanjing Agricultural University, No.1 Weigang, Nanjing, 210095 China

**Keywords:** *Flavobacterium* sp. NAU1659, *Magnaporthe oryzae*, β-1,6-glucanase *Fl*Glu30, Pustulan, Antifungal, Fungal cell wall

## Abstract

**Abstract:**

β-1,6-Glucan plays a crucial role in fungal cell walls by linking the outer layer of mannoproteins and the inner layer of β-1,3-glucan, contributing significantly to the maintenance of cell wall rigidity. Therefore, the hydrolysis of β-1,6-glucan by β-1,6-glucanase directly leads to the disintegration of the fungal cell wall. Here, a novel β-1,6-glucanase *Fl*Glu30 was identified from the endophytic *Flavobacterium* sp. NAU1659 and heterologously expressed in *Escherichia coli* BL21 (DE3). The optimal reaction conditions of purified *Fl*Glu30 were 50℃ and pH 6.0, resulting in a specific activity of 173.1 U/mg using pustulan as the substrate. The hydrolyzed products of *Fl*Glu30 to pustulan were mainly gentianose within 1 h of reaction. With the extension of reaction time, gentianose was gradually hydrolyzed to glucose, indicating that *Fl*Glu30 is an endo-β-1,6-glucanase. The germination of *Magnaporthe oryzae* Guy11 spores could not be inhibited by *Fl*Glu30, but the appressorium formation of spores was completely inhibited under the concentration of 250.0 U/mL *Fl*Glu30. The disruptions of cell wall and accumulation of intracellular reactive oxide species (ROS) were observed in *Fl*Glu30-treated *M. oryzae* Guy11 cells, suggesting the significant importance of β-1,6-glucan as a potential antifungal target and the potential application of *Fl*Glu30.

**Key points:**

*• β-1,6-Glucan is a key component maintaining the rigid structure of fungal cell wall.*

*• β-1,6-Glucanase is an antifungal protein with significant potential applications.*

*• FlGlu30 is the first reported β-1, 6-glucanase derived from Flavobacterium.*

## Introduction

The fungus *Magnaporthe oryzae* is the cause of rice blast, a highly destructive disease that severely impacts rice cultivation worldwide (Jin et al. 2024). Currently, efforts to control and manage rice blast include the development of resistant rice varieties, cultural practices and fungicide applications (Asibi et al. 2019). The breeding of resistant rice varieties involves a lengthy and resource-intensive process; therefore, chemical agents remain the primary means of controlling rice blast. However, with increasing concern about the environmental problems caused by chemical agents, researchers have begun to use more environmentally friendly biological control methods as alternatives (Haas and Defago 2005; Ye et al. 2020a,b).

Fungal cell walls are vital for cell integrity, as they provide mechanical protection from environmental stress (Gow et al. 2017). The fungal cell walls contain approximately 50–60% glucans, with β-1,6-glucan accounting for around 10% of the total glucan content (Ruiz-Herrera and Ortiz-Castellanos 2019). Serving as a cross-linking agent, β-1,6-glucan enhances the rigidity of the cell wall structure (Ye et al. 2023). Therefore, the degradation of β-1,6-glucan by β-1,6-glucanase can diminish the mechanical resilience of the cell walls, ultimately resulting in the lysis of fungal cells (Li et al. 2019; Ye et al. 2023). Additionally, these enzymes may also be involved in regulating fungal diseases (Fayad et al. 2001; Yamamoto et al. 1974).

β-1,6-Glucanase (EC 3.2.1.75) is an enzyme that specifically targets and hydrolyzes β-1,6-glycosidic bonds present in various glucans (Plakys et al. 2024; Wang et al. 2017). β-1,6-Glucanases derived from eukaryotic or prokaryotic are classified into glycoside hydrolase (GH) families 5 and 30 in the CAZy database (http://www.cazy.org/) based on their amino acid sequences (Wang et al. 2017). The GH30 family of β-1,6-glucanases utilizes an acid–base mechanism to hydrolyze β-glycoside bonds (Rezaie et al. 2018). This mechanism necessitates the presence of a minimum of two amino acid residues in the enzyme’s active site, with one serving as an acid or base and the other functioning as a nucleophile (Park et al. 2000). These enzymes belong to the glycosyl hydrolase A family, characterized by their three-dimensional triosephosphate isomerase barrel structure. Typically, their active site contains two glutamic acid residues, and the enzymes primarily target substrates within the cell wall structures of filamentous fungi and yeasts (Rast et al. 2003). In addition, substrates of β-1,6-glucanases can also be found as secretory or storage polysaccharides, such as pustulan (β-1,6-glucan) and laminarin (β-1,3–1,6-glucan), in certain fungi and lichens (Pradeep and Edison 2022; Tupe et al. 2022).

β-1,6-Glucanases derived from the GH5 family have hardly been reported for antifungal properties. However, researchers have found that β-1,6-glucanase *Vf*Glu1 derived from the GH5 family plays a key role in the infection of *Agaricus bisporus* by *Verticillium fungicola* (Amey et al. 2003). In addition, it has been reported that β-1,6-glucanases (EC 3.2.1.75) of some fungi are important for fungal mycoparasitism and probably cell wall cycling (Aspeborg et al. 2012). Therefore, we speculate that the β-1,6-glucanase sourced from the GH5 family may possess antifungal activity, but this requires more direct experimental verification.

In this study, the gene encoding β-1,6-glucanase *Fl*Glu30, belongs to GH30 family, was cloned from *Flavobacterium* sp. NAU1659 and heterologously expressed in *Escherichia coli* BL21(DE3). The recombinant β-1,6-glucanase *Fl*Glu30 was purified using Ni^2+^-NTA and exhibited maximal activity with pustulan. Consequently, the enzymatic properties of *Fl*Glu30 and its antifungal effects on *M. oryzae* Guy11 were investigated, respectively. Finally, the integrity of cell wall and accumulation of reactive oxygen species (ROS) in *Fl*Glu30-treated Guy11 cells were analyzed to understand the antifungal mechanisms of *Fl*Glu30. This is the first report of a β-1,6-glucanase from endophytic bacteria, laying the theoretical foundation for the application of β-1,6-glucanase in the biological control of plant pathogenic fungi.

## Materials and methods

### Strains, plasmids and reagents

Peptone and yeast extract were purchased from Oxoid Co. Ltd. (Beijing, China). All molecular biology reagents were purchased from TaKaRa Co., Ltd. (Otsu, Japan). Pustulan (Elicityl, Crolles, France) were purchased from Shanghai ZZBIO Co., Ltd. (Shanghai, China). Laminarin (from *Laminaria digitata*), pachyman, xylan, and carboxymethyl cellulose-sodium salt (CMC) were purchased from Sigma-Aldrich (St. Louis, MO, USA).

*Flavobacterium* sp. NAU1659, which was isolated from cucumbers’ rhizosphere soil and maintained in our lab, and *E. coli* BL21(DE3), purchased from Takara Bio Company (Kusatsu, Japan), were cultured in Luria–Bertani (LB) medium containing 10 g/L tryptone, 5 g/L yeast extract and 10 g/L NaCl at 30 ℃ and 37 ℃, respectively. *M. oryzae* strain Guy11 (ATCC 201236) was cultured in complete medium (CM) with or without 1.5% agar at 25 ℃ for 3–5 days. The plasmid pET-29a ( +), which was used for cloning and expressing of β-1,6-glucanase *Fl*Glu30 (GenBank accession number: PP690410), was obtained from Vazyme (Nanjing, China). The genomic DNA of strain NAU1659 was extracted following a previous report (Ye et al. 2022).

### Gene cloning, heterologous expression and purification of β-1,6-glucanase *Fl*Glu30

The signal peptide of *Fl*Glu30 was predicted using the Signal-3L 3.0 server (http://www.csbio.sjtu.edu.cn/bioinf/Signal-3L/). The coding regions corresponding to the gene, excluding the signal peptide, were amplified from the extracted DNA using the primer pairs *Fl*Glu30-F (5′-TAAGAAGGAGATATACATATGTCAAAAA ATGTTACTGCCAATTC-3′) and *Fl*Glu30-R (5′-GTGGTGGTGGTGGTGGT GCTCGAGTTACCAACGAAAGTAGCAACTGC-3′). The products were ligated to the *Nde*I/*Xho*I digested expression vector pET-29a, purchased from Takara Bio Company (Kusatsu, Japan), to generate the recombinant plasmid pET-29a-*Fl*Glu30. The ClonExpress II/One Step Cloning Kit (Vazyme, Nanjing, China) was employed for this purpose.

The recombinant plasmid was then introduced into *E. coli* BL21(DE3), and the transformed cells were screened and verified through DNA sequencing. Positive transformants were selected and cultured at 37 °C in LB medium supplemented with 100 μg/mL kanamycin until the optical density (OD600) reached 0.6–0.8. Subsequently, the culture medium was supplemented with isopropyl-β-D-1- thiogalactopyranoside (IPTG) at a final concentration of 0.2 mM to induce protein expression and further incubated at 16 °C for 20 h.

The cells harvested from the induced culture were suspended in 50 mM PBS buffer (pH 6.0) and disrupted by ultrasonication (Sonicator 201 M, Kubota, Osaka, Japan). The mixture of disrupted cells was centrifuged at 12,000 rpm for 10 min at 4 °C to separate into soluble and insoluble fractions. The recombinant fusion proteins, tagged with a C-terminal 6His-tag, were purified using Ni^2+^-nitrilotriacetic acid (Transgen, Beijing, China) resin following the manufacturer’s instructions. Then, the purified *Fl*Glu30 protein was collected and dialyzed overnight at 4 °C against 50 mM PBS buffer (pH 6.0) to remove imidazole. Protein purity was determined using SDS-PAGE, and protein concentration was assessed using the Bradford method (Bradford 1976).

### Enzyme characteristics of β-1,6-glucanase *Fl*Glu30

The activity of the purified recombinant enzyme was assessed by measuring the release of reducing sugars from a pustulan solution (5 mg/mL). The reaction mixture contained 60 μL of the pustulan and 5 μg/mL of purified *Fl*Glu30 was incubated at 50 °C for 10 min. Subsequently, an equal volume of the 3,5-dinitrosalicylic acid reagent was added to the reaction and incubated at 100 °C for 10 min (Gusakov et al. 2011). One unit of enzyme activity was defined as the quantity of enzyme needed to liberate reducing sugars equivalent to 1 μmol of glucose per minute under the specified testing conditions.

The characteristics of β-1,6-glucanase *Fl*Glu30 were analyzed using 0.5% pustulan as the substrate. The optimal temperature for β-1,6-glucanase *Fl*Glu30 activity was determined within a range of 20–80 °C, with intervals of 10 °C, in a 50 mM PBS buffer at pH 6.0. To assess the thermal stability of the enzyme, the enzyme solution was incubated at different temperatures (4 °C, 20 °C, 30 °C, 40 °C, 50 °C, 60 °C, 70 °C and 80 °C) for 1 h, 2 h, 4 h and 8 h. After the respective incubation periods, 0.5% pustulan was added to the enzyme solution at 50 °C for a 10 min. The residual hydrolytic activity of the enzyme was measured. The initial enzyme activity of *Fl*Glu30 that was not subjected to incubation at different temperatures was used as a reference to calculate the relative enzyme activity after incubation. *Fl*Glu30 exhibited the most favorable pH at 50 °C by evaluating its performance across different buffers: 50 mM sodium acetate buffer at pH 4.0–6.0, 50 mM Tris–HCl buffer at pH 6.0–9.0, 50 mM PBS buffer at pH 6.0–8.0 and 50 mM glycine–NaOH buffer at pH 9.0–11.0. To evaluate its pH stability, the activity of *Fl*Glu30 was measured under standard conditions after incubating for 24 h at 4 °C in the aforementioned buffers without substrate.

The enzymatic activity of *Fl*Glu30 was evaluated for its susceptibility to potential inhibitors or activators. This was accomplished by introducing a concentration of 1 mM of various metal salts (Ni^2+^, Ba^2+^, Mg^2+^, Zn^2+^, Fe^2+^, Co^2+^, Na^+^, Al^3+^, Ca^2+^, Mn^2+^, Cu^2+^, K^+^, Cr^3+^) and other chemical agents at various concentrations (methanol, ethanol, isopropanol, acetone, acetonitrile, ethylene diamine tetraacetic acid (EDTA), dimethyl sulfoxide (DMSO), Tween 80, Triton X-100, β-mercaptoethanol (β-ME), urea, dithiothreitol (DTT), phenylmethanesulfonyl fluoride (PMSF)) into the reaction mixture. Following 1 h of incubation at 40 °C, the residual activity was measured at 50 °C for 10 min after supplement with 0.5% pustulan.

To determine the substrate specificity of *Fl*Glu30, the enzyme activity was measured in 50 mM PBS buffer (pH 6.0) containing 5 mg/mL of each substrate, including pustulan (β-1,6-glucan), yeast glucan (β-1,3–1,6-glucan), laminarin (β-1,3–1,6-glucan), pachyman (β-1,3-glucan), xylan (β-1,4-glucan), and CMC (β-1,4-glucan). The reaction suppled with inactive *Fl*Glu30 used as the control. In addition, the different concentrations of pustulan ranging from 1 to 10 mg/mL mixed with purified *Fl*Glu30 under the optimal conditions for 10 min were used to measure the kinetic constants of *Fl*Glu30. The kinetic rate constants, K_m_ and V_max_, were obtained by examining the data using a Lineweaver–Burk plot (Dowd and Riggs 1965).

### Analysis of the hydrolysis products

Purified *Fl*Glu30 (10 μg) was added to reaction mixture containing 0.5% pustulan and then incubated at 50 ℃ for different time (1 min, 5 min, 10 min, 30 min, 1 h, 2 h, 4 h, 8 h, 12 h, 16 h, and 24 h) in 50 mM PBS buffer (pH 6.0). After incubation, the reaction mixtures were promptly boiled at 100 ℃ for 10 min and centrifuged at 12,000 rpm for 3 min. Subsequently, the released oligosaccharides were analyzed using thin-layer chromatography (TLC). The samples were spotted onto a TLC plate, developed in *n*-butanol/acetic acid/water (2:1:1, v/v/v) as a solvent, and then sprayed with sulfuric acid/methanol (1:1). The products were displayed after heating the sheet at 95 ℃ in an oven for 5 min.

### Inhibitory effect of β-1,6-glucanase *Fl*Glu30 on spore germination of Guy11

For spore production, the mycelia were cultured on a corn agar medium (SDC) containing 100 g of rice straw, 40 g of corn powder, and 15 g of agar dissolved in 1 L of deionized water. Subsequently, the plates containing mycelia were incubated at a temperature of 28 °C for 3 days in a dark environment. Then, the aerial hyphae were scraped from the culture, and the plates were subjected to continuous illumination under light for an additional 3 days (Qi et al. 2016).

The conidia were washed with ddH_2_O, and passed through three-layer lens paper to filter out hyphae. Then, the conidia were collected by centrifugation at 5000 rpm for 5 min and re-suspended in 200 μL of 50 mM PBS buffer (pH 6.0). Purified *Fl*Glu30 was sterilized though a 0.22 μm pore-size filter. Finally, the conidia were mixed with different concentration of purified *Fl*Glu30 and spotted on a cover glass (12542B, Fisherbrand, ThermoFisher, Waltham, MA, USA) to calculate the germination rate of conidia and appressorium formation under a microscopic (CX23, Olympus, Tokyo, Japan). The final concentration of conidia in mixtures was 5 × 10^4^ cells/mL using a hemocytometer (Wang et al. 2021). The heat-inactivated *Fl*Glu30 was used as control.

### Analysis of cell membrane integrity, reactive oxygen species (ROS) and chitin content of *Fl*Glu30-treated Guy11 cells

The detections of cell membrane integrity, ROS and chitin content of *Fl*Glu30 treated Guy11 cells were performed following previous description with minor modifications (Ye et al. 2023). The final concentration of 5 × 10^4^ conidia/mL Guy11 cells were incubated with 160 U/mL *Fl*Glu30 at 28 ℃ for 1.0 and 3.0 h, respectively. The cells were washed and re-suspended in a 50 mM PBS buffer at pH of 6.0 after *Fl*Glu30 treatment. Subsequently, 5.0 μM propidium iodide (PI) and 10 mg/mL of calcofluor white (CFW) were added and incubated for another 20 min and 5 min in darkness, respectively. Moreover, *Fl*Glu30-treated conidia were mixed with 50 μM 2′,7′-dichlorodihydrofluorescein diacetate (H_2_DCFDA) for a duration of 20 min at room temperature to analysis ROS content. The spores were washed with PBS buffer prior to the mentioned staining steps to ensure cleanliness, and after staining, any residual dye was washed off using the same buffer solution. All images were captured and visualized using a confocal laser scanning microscopy (CLSM) (Leica TCS SP8, Wetzlar, Germany).

### Statistical analysis

All analyses and measurements were performed in triplicate. The values are represented by the mean ± standard deviation (SD). Single-factor analysis of variance (one-way ANOVA) and Duncan’s multiple range test were carried out using SPSS statistics software version 22.0 (IBM Corporation, Armonk, NY, USA) to evaluate the significant differences (*P* ≤ 0.05) between the different treatments.

## Results

### Identification of the β-1,6-glucanase *Fl*Glu30 from *Flavobacterium* sp. NAU1659

A novel β-1,6-glucanase *Fl*Glu30 from *Flavobacterium* sp. NAU1659 was identified by searching nucleotide sequence in the NCBI database. The complete coding sequence of *Fl*Glu30 consists of 1425 base pairs that encode for 474 amino acids. The protein has a calculated molecular weight of 52.2 kDa and a pI of 7.6. Analysis of the Signal-3L 3.0 server revealed the presence of a signal peptide (MKNINKKLQILVLLPLIAM QLNCGS) at the N-terminal region of the protein.

**Fig. 1:**
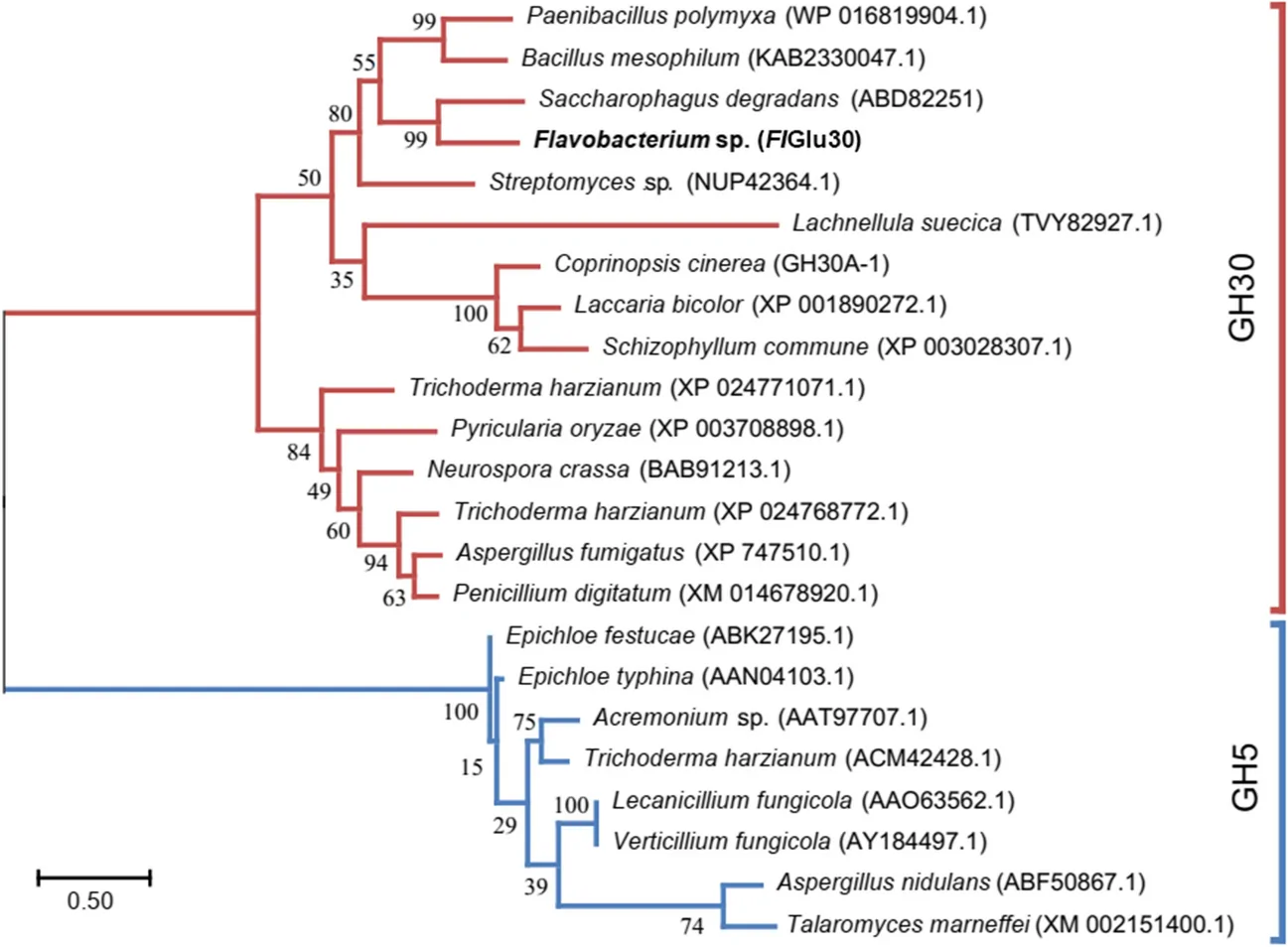
Phylogenetic analysis of β-1,6-glucanase from various sources. The distances were determined and the phylogenetic tree was constructed using the neighbor-joining algorithm based on the amino acid sequence alignment in MEGA7 (Kumar et al. 2016). Bootstrap values based on 1000 replications are listed as percentages at branch points. Bar, 0.1 substitutions per amino acid position. GH30 and GH5 represent glycoside hydrolase family 30 and 5, respectively. β-1,6-Glucanase *Fl*Glu30 from *Flavobacterium* sp. NAU1659 was represented in bold black font

As shown in Fig. 1, *Fl*Glu30 was aligned with other β-1,6-glucanases from the GH5 and GH30 family including fungal and bacterial enzymes. The phylogenetic analysis of the β-1,6-glucanases amino acid sequence showed that *Fl*Glu30 is a member of the GH30 family and closely related to the bacterial β-1,6-glucanases. In the list provided, the proteins identified from *Saccharophagus degradans* (ABD82251, 49.3%), *Bacillus mesophilum* (KAB2330047.1, 39.8%), and *Paenibacillus polymyxa* (WP016819904.1, 38.9%) exhibited the highest homology to *Fl*Glu30. The similarity between other β-1,6-glucanase in the GH30 family and *Fl*Glu30 ranges from 27.2 to 36.4%.

**Fig. 2:**
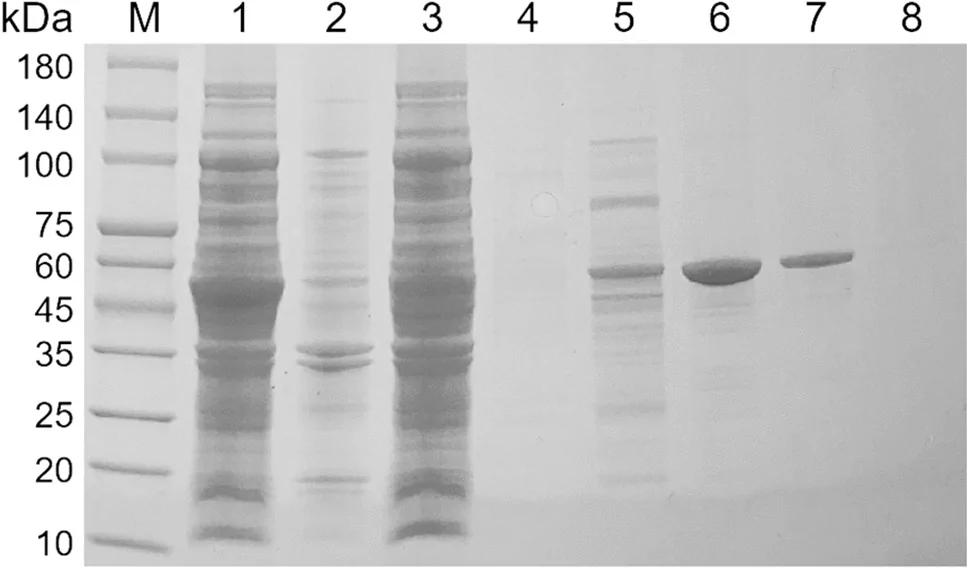
SDS-PAGE analysis of the recombinant *Fl*Glu30 protein. Purified *Fl*Glu30 protein was loaded onto a 12.0% Tris–glycine SDS-PAGE gel and were stained with Coomassie brilliant blue R-250. M: protein low molecular weight marker; lane 1: The cell disruption supernatant of *E. coli* BL21(DE3) containing pET-29a-*Fl*Glu30 recombinant plasmid; lane 2: The cell disruption precipitate of *E. coli* BL21(DE3) containing pET-29a-*Fl*Glu30 recombinant plasmid; lane 3: flow-through of cell disruption supernatant after binding to the Ni^2+^ column; lane 4: the eluent solution after the Ni^2+^ column washed with 20 mM Tris–HCl buffer; lane 5–8: different purities of *Fl*Glu30 protein after washing with 20 mM Tris–HCl buffer containing 50, 100, 200 and 300 mM imidazolium, respectively

The purification process and harvest efficiency of recombinant *Fl*Glu30 protein were showed in Table 1. Through a two-step purification process, *Fl*Glu30 was purified 20.48-fold, resulting in a 34.64% recovery and a specific activity of 173.12 U/mg using pustulan as a substrate. After that, SDS-PAGE was conducted to determine the apparent molecular mass of the recombinant protein. The purified *Fl*Glu30 protein exhibited a cleared band on SDS-PAGE, with an approximate molecular weight of around 50 kDa (Fig. 2).

**Table 1:** The analysis of purification efficiency of *Fl*Glu5 protein

Purification	Total protein (mg)	Total activity (U)	Specific activity (U/mg)	Purification fold	Yield (%)
Crude enzyme	130.8	849.4	6.5	1	100
Ni^2+^-NTA	1.7	294.2	173.1	26.7	34.6

### Effects of pH and temperature on recombinant FlGlu30 activity and stability

**Fig. 3:**
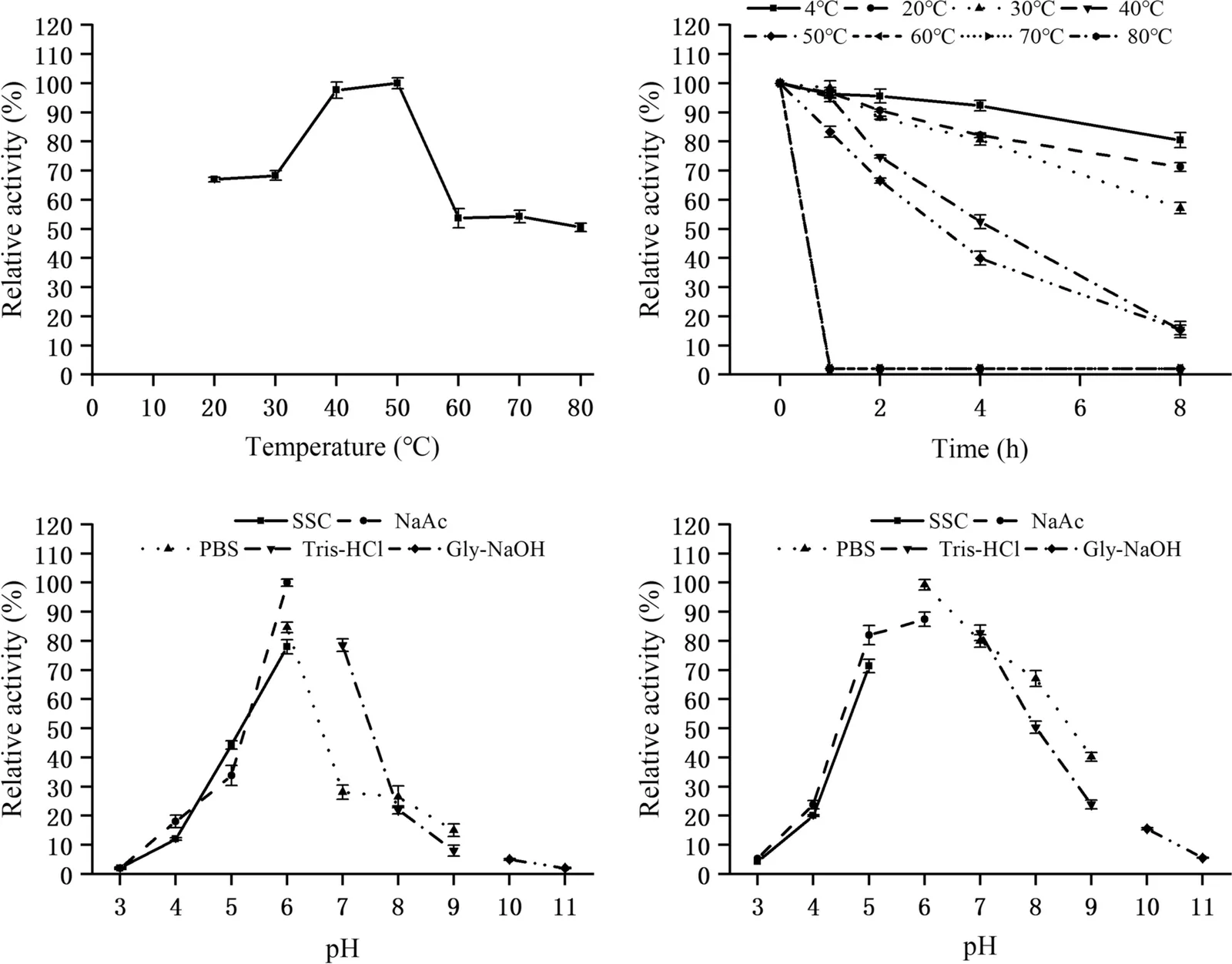
Effects of temperature and pH on the activity and stability of *Fl*Glu30. (a) Determination of the optimal temperature of *Fl*Glu30. Activity was measured in 50 mM PBS buffer (pH 6.0) at 20–70 ℃ for 10 min. (b) Thermostability of purified *Fl*Glu30. The residual activity was measured under optimal conditions after incubation of the enzyme at the indicated temperatures for 1–8 h. (c) Determination of the optimal pH of *Fl*Glu30. Assays were carried out with 5 mg/mL pustulan as substrate at 50 ℃ for 10 min in buffers with varying pH (pH 3.0–11.0). (d) pH stability of purified *Fl*Glu30. The residual enzyme activity was measured under optimal conditions after incubation of the purified enzyme in buffers with various pH values at 4 ℃ for 24 h

The enzymatic properties of *Fl*Glu30 were determined utilizing pustulan as the substrate. The results showed that purified *Fl*Glu30 exhibited clear optimum pH and temperature were 6.0 and 50 °C, respectively (Fig. 3a and c). Moreover, *Fl*Glu30 exhibited excellent stability at 50 ℃, retaining over 80% of its activity after 1 h. About more than 50% of the enzyme activity was still detected after 8 h of incubation under below 30 ℃ (Fig. 3b). However, a notable decline in enzymatic stability was observed after 1 h when the temperature exceeded 60 ℃, indicating β-1,6-glucanase *Fl*Glu30 could be classified as a mesophilic enzyme. The purified *Fl*Glu30 demonstrated a high level of activity within the pH range of 5.0 to 6.0, but showed minimal activity at pH values above 7.0 or below 5.0 (Fig. 3c). The enzyme retained more than 70% of its initial activity at pH 5.0 to 7.0 after incubation for 24 h. However, its stability decreased significantly at pH values below 5.0 or above 7.0 (Fig. 3d).

### Effect of metal ions and chemical agents on the enzymatic activity of *FlG*lu30

To analyze the effect of different metal ions or chemical reagents on *Fl*Glu30 activity, the residual activities of the enzymes were measured following 1 h of incubation with 1 mM concentrations of various metal ions or chemical reagents, respectively. The results showed that K^+^ and Na^+^ had almost no effect on β-1,6-glucanase activity of *Fl*Glu30 at the tested concentration (Table 2). Mg^2+^ and Mn^2+^ were found to have a slight stimulating effect on *Fl*Glu30 activity, resulting in approximately 20% increase. However, the activity of *Fl*Glu30 was significantly inhibited by Fe^3+^ and Zn^2+^. Additionally, it was observed that high concentrations of EDTA (10 mM), ethanol, isopropanol, acetone and acetonitrile completely inhibited the glucosidase activity of *Fl*Glu30. Methanol and PMSF treatment resulted in approximately a 60% decrease in *Fl*Glu30 activity. By contrast, Tween 80 and β-ME slightly improved the β-1,6-glucanase activity, whereas EDTA (1 mM), DMSO, Triton X-100, urea, and DTT had no significant effect.

**Table 2:** Effects of metal ions and chemical agents on *Fl*Glu5 activity

	Reagent^a^	Concentration	Relative activity (%)
Metal ions	No addition	1 mM	100.00 ± 2.62
	K^+^	1 mM	101.86 ± 1.07
	Na^+^	1 mM	108.12 ± 1.42
	Ba^2+^	1 mM	115.82 ± 0.78
	Co^2+^	1 mM	112.20 ± 1.22
	Cu^2+^	1 mM	112.20 ± 1.82
	Fe^3+^	1 mM	46.55 ± 2.05
	Mg^2+^	1 mM	121.26 ± 2.75
	Mn^2+^	1 mM	120.18 ± 1.60
	Ni^2+^	1 mM	72.76 ± 0.96
	Zn^2+^	1 mM	44.11 ± 2.85
	Cr^2+^	1 mM	79.19 ± 3.06
	Ca^2+^	1 mM	114..19 ± 2.89
Chemical agents	Methanol	20%	39.08 ± 2.35
	Ethanol	20%	ND
	Isopropanol	20%	ND
	Acetone	20%	ND
	Acetonitrile	20%	ND
	EDTA	1 mM	82.11 ± 1.76
	EDTA	5 mM	66.58 ± 0.46
	EDTA	10 mM	3.27 ± 0.56
	DMSO	5%	91.40 ± 2.78
	Tween 80	1%	113.16 ± 1.39
	Triton X-100	1%	95.44 ± 1.88
	β-ME	5 mM	114.34 ± 2.13
	Urea	5 mM	103.43 ± 2.21
	DTT	5 mM	89.17 ± 0.97
	PMSF	5 mM	38.92 ± 1.36

a, all metals were provided as chloride salts; b, ND indicated that no activity was detected

### Substrate specificity and analysis of the hydrolysis products

After conducting tests on polysaccharide substrates with different linkages, it was discovered that *Fl*Glu30 enzyme effectively cleaved and liberated soluble glucose from pustulan. However, *Fl*Glu30 showed no hydrolytic activity towards laminarin, which also contains β-1,6-glycosidic linkages, indicating potential substrate selectivity of the enzyme. Additionally, *Fl*Glu30 exhibited no hydrolytic activity towards polysaccharides containing β-1,3 or β-1,4-glycosidic linkages, such as pachyman, xylan, and cellulose. Under the optimal conditions, the Michaelis–Menten constant (*K*_m_) and maximum reaction rate (*V*_max_) were found to be 4.2 mg·mL^−1^ and 318.1 μmol·min^−1^·mg^−1^, respectively. The above results suggested that pustulan, which solely contains β-1,6-glycosidic linkages, was the optimal substrate for *Fl*Glu30. Therefore, we have confirmed that *Fl*Glu30 functions as a β-1,6-glucanase.

**Fig. 4:**
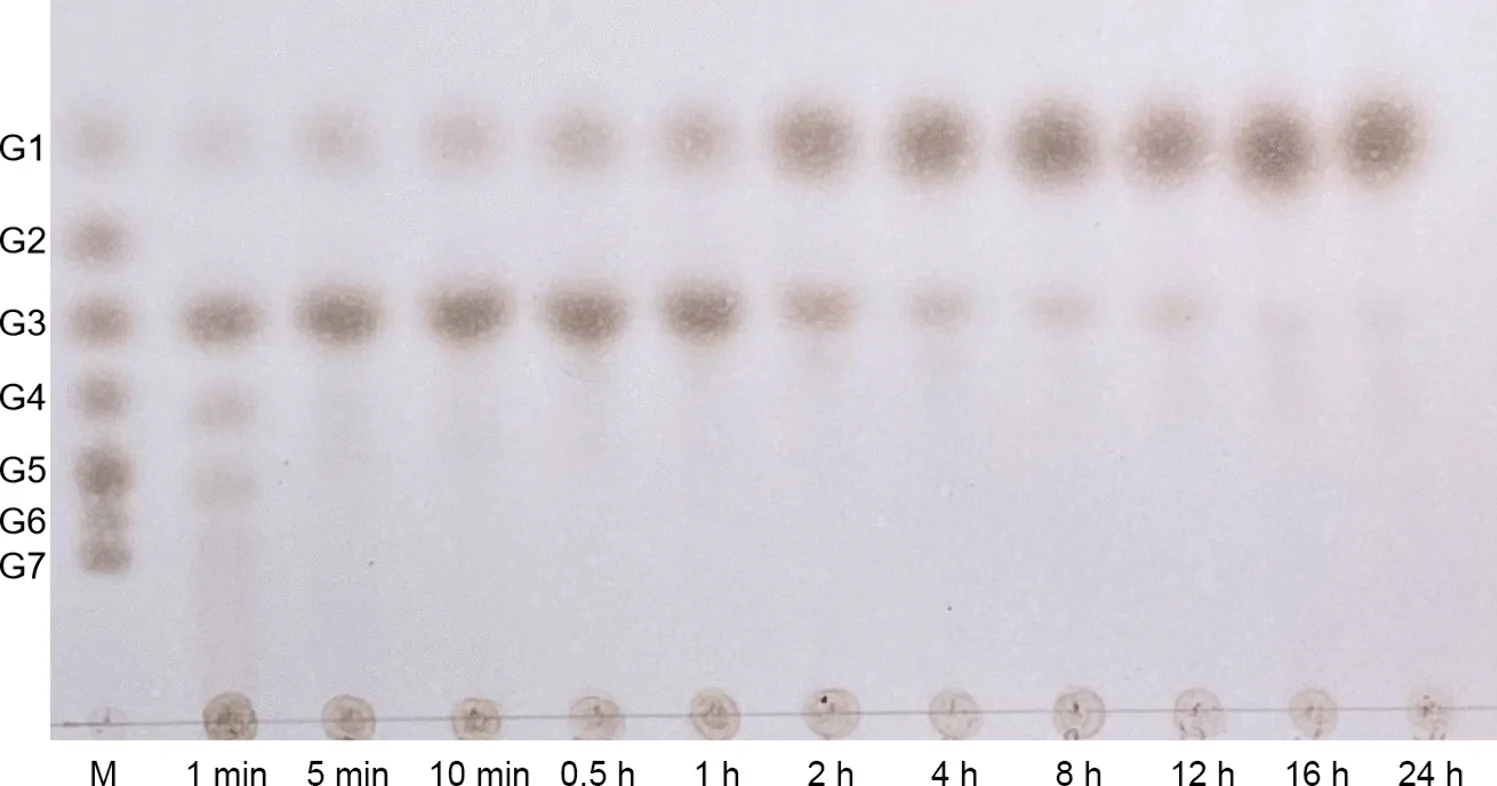
Analysis of the hydrolysates of *Fl*Glu30 with pustulan. TLC analysis of hydrolysis products produced by incubation of *Fl*Glu30 with 0.5% pustulan for various times. Lane M represents malto-oligosaccharides of different degrees of polymerization

Subsequently, the hydrolytic properties of *Fl*Glu30 were investigated using pustulan (0.5%, w/v) as substrate by utilizing thin-layer chromatography (TLC). The results showed that after incubation of *Fl*Glu30 with the pustulan for 1 min, the main product is gentianose, but there were also small amounts of oligosaccharides with chain lengths ≥ 4 generated. Throughout the entire reaction process, the content of gentianose initially increased and then decreased, ultimately being hydrolyzed by *Fl*Glu30 into monosaccharides (Fig. 4). Therefore, we speculated that *Fl*Glu30 was an endo-β-1,6-glucanase.

### Effects of *Fl*Glu30 on spores’ germination and appressorium formation

**Fig. 5:**
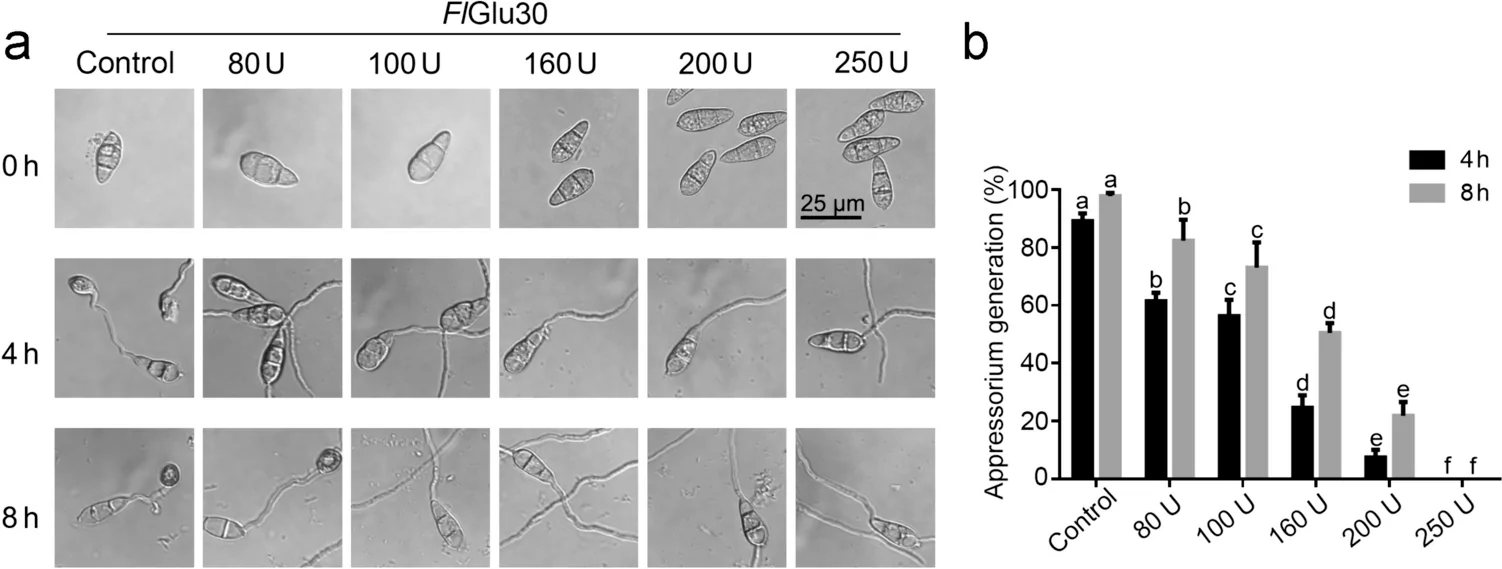
Effects of *Fl*Glu30 on the formation of germ tubes and appressoria. β-1,6-Glucanase *Fl*Glu30 was added to the spores’ suspension for determine the effects of the enzyme on fungal germination (**a**). The number of germinated spores and appressorium formation was counted after treatment with *Fl*Glu30 for different times (**b**). Treatments with heat-inactivated *Fl*Glu30 were used as the control

The inhibitory effect of *Fl*Glu30 on *M. oryzae* Guy11 was determined by measuring the rate of spores’ germination and appressorium formation. The results showed that *Fl*Glu30 could not inhibit the germination of spores, but could significantly inhibit the formation of appressorium (Fig. 5). Following an 8-h treatment with 200.0 U/mL *Fl*Glu30, the appressorium formation rate of conidia was decreased significantly from 97.6% in control group to 17.3% in treatment group (Fig. 5b). Moreover, almost all spores’ germination were observed within 2 h after treatment with higher concentration of *Fl*Glu30 (500.0 U/mL) (data not shown), suggesting that the low hydrolysis efficiency of *Fl*Glu30 on the fungal cell wall may not inhibit the germination of spores. However, these findings highlight the potential significance of β-1,6-glucan in the fungal cell wall as a key target for impeding fungal growth.

### Treatment with *Fl*Glu30 resulted in the disruption of the cell membrane integrity in Guy11 cells

**Fig. 6:**
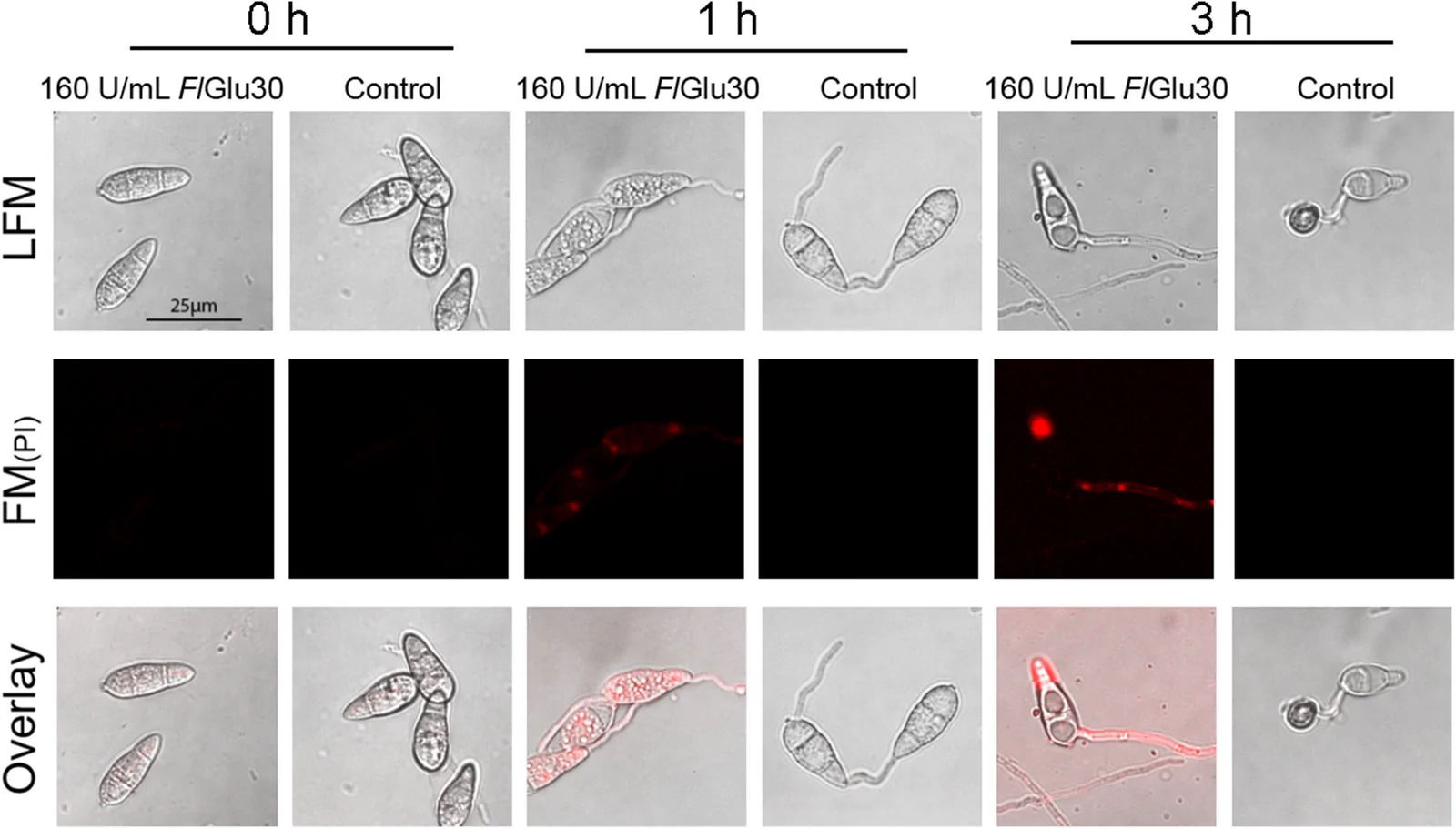
Detection of cell membrane integrity after conidia treated with *Fl*Glu30. After exposure to *Fl*Glu30, the cell membrane integrity was detected by 5 μM PI staining at different time. LFM, light-field microscopy; FM, fluorescence microscopy

In order to gain further insights into the mechanisms underlying *Fl*Glu30’s ability to inhibit appressorium formation, this study focused on evaluating the integrity of cell membranes, ROS accumulation, and chitin distribution within the cells. The intact cell membrane of a living cell is selectively permeable (McElhaney 1975). Therefore, propidium iodide (PI) cannot normally pass through the cell membrane and bind to DNA in the cells, however, it can enter cells with damaged cell membranes (Ye et al. 2023). By binding to the DNA of necrotic cell, propidium iodide (PI) exhibited red fluorescence under the fluorescence field, allowing us to identify impaired cell membranes based on the fluorescent color. Here, the results showed that after 1 h of treatment with *Fl*Glu30, the mycelial cells showed red bright spots under the fluorescence field, indicating that PI entered the fungal cells and bound with DNA (Fig. 6). In addition, with the prolongation of treatment time, both of the proportion of mycelia containing fluorescent and the fluorescence intensity of hyphae were increased, indicating that hydrolysis of the fungal cell wall by *Fl*Glu30 caused damage to Guy11 cells’ membranes (Fig. 6).

### Treatment with *Fl*Glu30 induced the burst of intracellular reactive oxygen species (ROS) in the cells

**Fig. 7:**
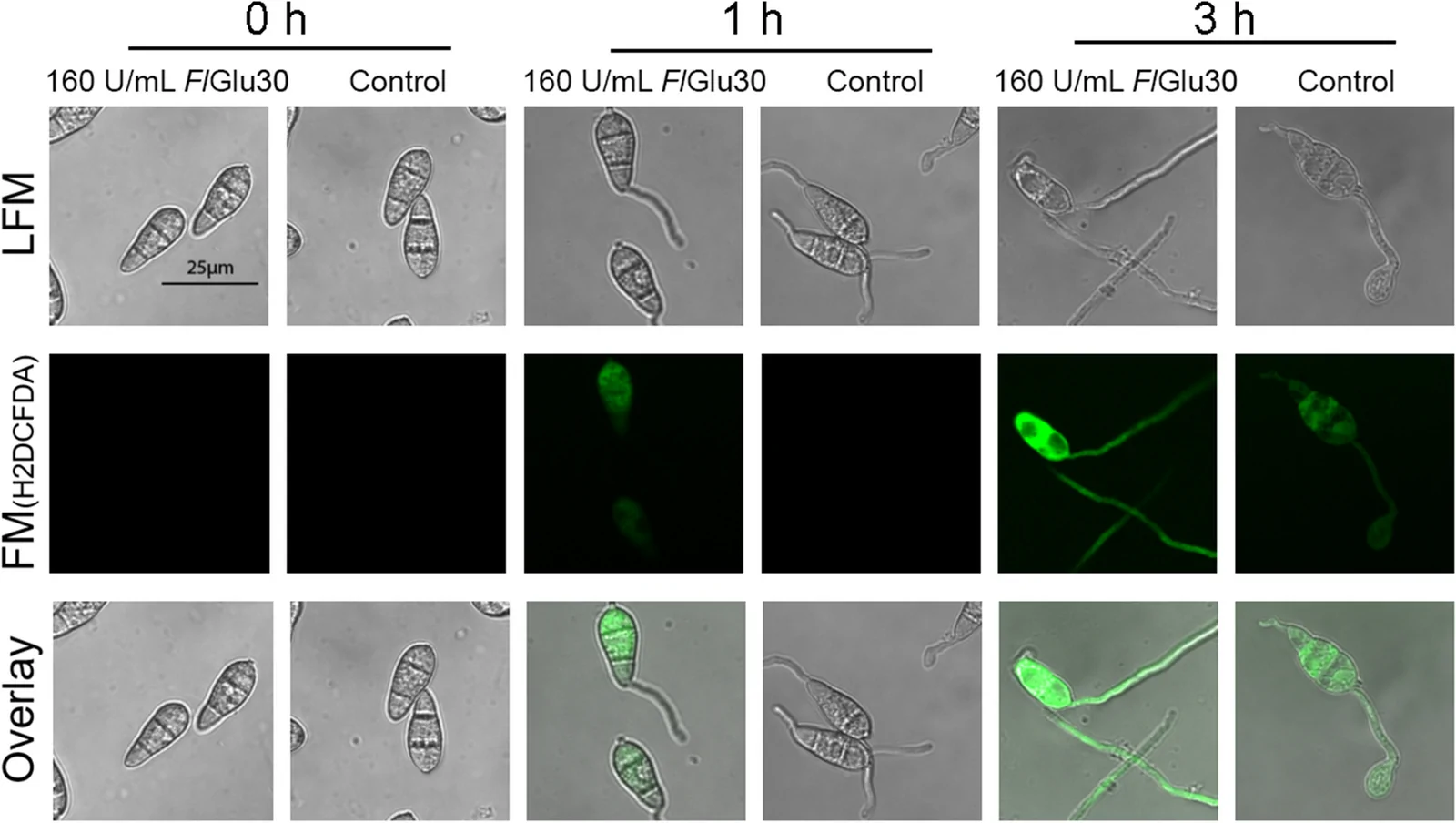
Detection of ROS accumulation after conidia treated with *Fl*Glu30. Detection of ROS was based on 50-μM H2DCFDA staining after conidia treated with *Fl*Glu30 for 0, 1, and 3 h, respectively. LFM, light-field microscopy; FM, fluorescence microscopy

The induction of cell wall synthesis and regulation of cell wall integrity (CWI) in fungi are associated with the production of ROS (Fuchs and Mylonakis 2009; Yu et al. 2016). Upon entering the cell, H2DCFDA is oxidized by intracellular ROS, resulting in the generation of the fluorescent chromophore. So, H2DCFDA was often used to analyze the accumulation of intracellular ROS under environmental stress. The results showed that compared with the control group, intracellular ROS began to accumulate after *Fl*Glu30 treatment for 1 h (Fig. 7). With the increase of treatment time, a small amount of ROS accumulated in the cells of control group after 3 h, but its content was significantly lower than that in *Fl*Glu30 treated cells, indicating the hydrolysis stress of *Fl*Glu30 on the fungal cell wall induced the burst of ROS (Fig. 7).

### The pathway of cell wall integrity (CWI) was activated in *Fl*Glu30-treated cells

**Fig. 8:**
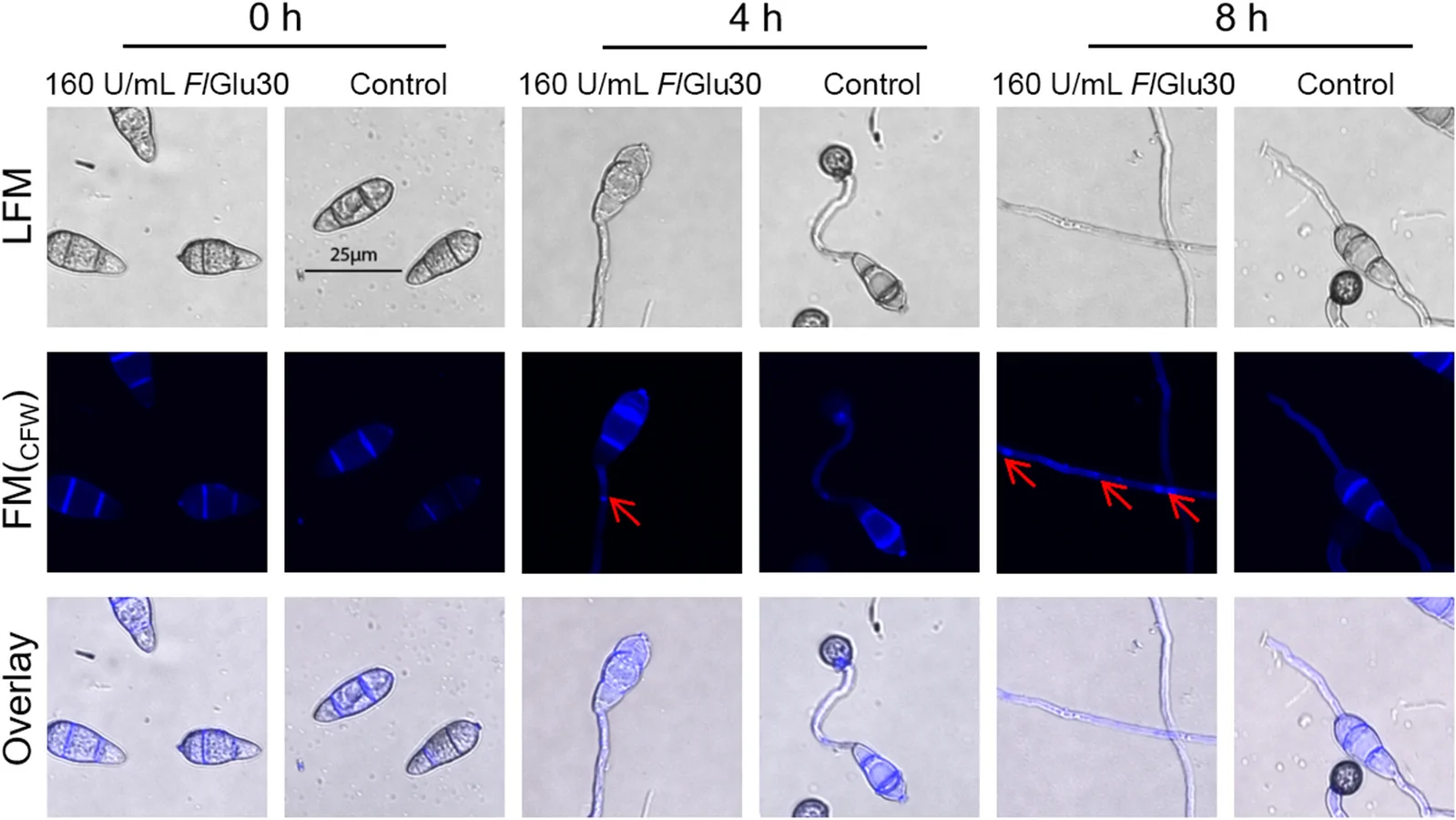
Mycelial calcofluor white (CFW) staining. CFW staining (10 mg/mL) was employed to analyze the chitin distribution in the cell wall after Guy11 cells treated with *Fl*Glu30. The degraded hyphae were indicated by red arrowheads. LFM, light-field microscopy; FM, fluorescence microscopy

The cell wall plays a vital role in preserving cell shape and safeguarding cells against harsh environmental conditions (Bermejo et al. 2008). When a component of the fungal cell wall is disrupted, it often triggers the fungal CWI pathway to increase the synthesis of other components to compensate for the damage caused by the absence of that component (Lu et al. 2023). Here, the CFW dye, which can specifically bind to chitin components in the fungal cell wall, is used to detect changes in the distribution of chitin in the cell wall after *Fl*Glu30 treatment of *M. oryzae* cells. The results revealed that, in contrast to the uniform distribution of chitin in the cell wall of the control group, the cell wall of *M. oryzae* Guy11 spores exhibited an uneven distribution of chitin after being treated with *Fl*Glu30 (Fig. 8). The same results were also reported in the *Fusarium oxysporum* cells treated with β-1,6-glucanase GluM (Ye et al. 2023).

## Discussion

Most of the reported β-1,6-glucanases are derived from fungi, where they play important roles in fungal cell wall remodeling and inhibition of the growth of other fungi. For example, three β-1,6-glucanases, BGN16.1, BGN16.2, and BGN16.3, have been identified in the antagonistic fungus *Trichoderma harzianum* (de la Cruz and Llobell 1999; de la Cruz et al. 1995). The most active β-1,6-glucanase derived from fungi was GH30A, which sourced from *Coprinopsis cinerea* ATCC56838, with a hydrolytic activity of 776.5 U/mg towards 1% pustulan (Liu et al. 2020). Furthermore, an increasing number of β-1,6-glucanases sourced from bacteria are being reported. Such as, the β-1,6-glucanase GluM from *Corallococcus* sp. EGB exhibited high hydrolytic activity of up to 24,000 U/mg towards yeast glucan (Li et al. 2019). Researchers have found that this enzyme plays a crucial role in the predation of *M. oryzae* and *F. oxysporum* by the myxobacteria strain EGB (Li et al. 2019; Ye et al. 2023). *Flavobacterium* is an important plant rhizosphere microorganism capable of secreting abundant extracellular glycoside hydrolases to degrade organic macromolecules in the plant rhizosphere (Enisoglu-Atalay et al. 2018). However, no β-1,6-glucanase sourced from microorganisms belonging to the *Flavobacterium* genus has been reported. Here, the enzyme characteristics and antifungal properties of the β-1,6-glucanase *Fl*Glu30 were studied and compared with the other reported β-1,6-glucanases. The findings enriched the repository of β-1,6-glucanases and lay the foundation for elucidating the potential ecological functions of *Flavobacterium* in the plant rhizosphere.

By utilizing fungal cell wall-degrading enzymes (CWDEs), such as chitinases, β-D-glucanases, chitosanases and proteases, bacteria are believed to exert their antifungal effects (Gutierrez-Gongora and Geddes-McAlister 2021; Takashima et al. 2023; Tue et al. 2024; Wang et al. 2021). These enzymes play a crucial role in the degradation of the structural components of fungal cell wall, effectively impeding the growth and proliferation of fungi (Ghasemi et al. 2020; Gutierrez-Gongora and Geddes-McAlister 2021). Furthermore, reducing the formation of appressoria on spores could significantly diminish the infections of rice by these spores, consequently lowering the incidence of rice blast disease (Huang et al. 2022; Shahriar et al. 2020). *Fl*Glu30, possessing endo-β-1,6-glucanase activity, has been shown in antifungal assays to display antagonistic effects against fungus *M. oryzae*.

Intracellular ROS play a critical role in activities of cellular life (Ye et al. 2023). Low concentrations of ROS can serve as intercellular messengers for many activities; however, a burst of ROS can lead to apoptosis or cell death (Cadenas and Davies 2000). Ye et al. (2023) reported that the β-1,6-glucanase GluM from the *Corallococcus* sp. EGB hydrolyzed the cell wall of *F. oxysporum*, leading to the burst of intracellular ROS and subsequent apoptosis of fungal cells.

Here, the purified *Fl*Glu30 enzyme has demonstrated promising efficacy in inhibiting the formation of appressoria by the Guy11 strain. The fungistatic effect of *Fl*Glu30, as revealed by staining with PI, H2DCFDA and CFW, can be attributed, at least in part, to its capacity for hydrolyzing the cell walls of *M. oryzae* Guy11 cells. Organisms utilize multiple signaling pathways to react to extracellular stimuli and uphold their cell wall integrity in response to changes in their environment (Fuchs and Mylonakis 2009). These pathways include processes such as ROS generation and cell wall remodeling. Therefore, the disruption of cell membrane integrity, accumulation of intracellular ROS and the alterations in chitin distribution in the cell wall induced by *Fl*Glu30 treatment on *M. oryzae* Guy11 suggesting that β-1,6-glucan could serve as a key target for the biological control of pathogenic fungi.

In conclusion, a novel β-1,6-glucanase *Fl*Glu30 gene was cloned from genome of *Flavobacterium* sp. NAU1659 and heterologously expressed in *E. coli* BL21(DE3). The optimum reaction temperature and pH of *Fl*Glu30 were 50℃ and 6.0, respectively. Under the optimum reaction conditions, the specific enzyme activity of *Fl*Glu30 with pustulan as substrate was 173.1 U/mL. The hydrolysis products, primarily consisting of gentianose, were detected at the beginning of the reaction, and with prolonged reaction time, pustulan was ultimately hydrolyzed into monosaccharides, indicating that *Fl*Glu30 is an endo-β-1,6-glucanase. The appressorium formation of spores of *M. oryzae* Guy11 was completely inhibited by 250.0 U/mL *Fl*Glu30. PI and H2DCFDA staining showed that hydrolysis of Guy11 cell wall by *Fl*Glu30 resulted in the disruptions of cell membrane and accumulation of intracellular ROS. This study provides a theoretical basis and genetic resources for the application of β-1,6-glucanase in the biological control of plant pathogenic fungi.

## Data Availability

The data that support the results of this research are available from the corresponding author.
